# Beyond the Science: Advancing the "Art and Craft" of Implementation in the Training and Practice of Global Health

**DOI:** 10.34172/ijhpm.2020.131

**Published:** 2020-07-14

**Authors:** Ejemai Amaize Eboreime, Aduragbemi Banke-Thomas

**Affiliations:** ^1^Department of Medicine, Faculty of Medicine and Dentistry, University of Alberta, Edmonton, AB, Canada.; ^2^Department of Planning, Research and Statistics, National Primary Health Care Development Agency, Abuja, Nigeria.; ^3^Department of Health Policy, London School of Economics and Political Science, London, UK.

**Keywords:** Implementation Science, Global Health, Mentorship, Apprenticeship, Training, Capacity Building

## Abstract

Interesting debates are ongoing on how to develop practical implementation science competencies that can bridge the "know-do" gap in global health. We advance these debates by arguing that apprenticeship and mentorship models drawn from "art and craft" used in industry is the missing piece of the puzzle that will bridge the persisting gap between academics and real-world practitioners. We propose examples of such models and how they can be applied to improve existing capacity building programs, as well as implementation in practice.

## Background

“*The significant problems we face cannot be solved at the same level of thinking we were at when we created them*”

 Albert Einstein

Strong debates are ongoing within academic spheres on how to develop implementation science approaches that are suitable to the field of global health. There are concerns that despite the huge investments in health programs in low- and middle-income countries (LMICs), the interventions are not yielding commensurate results. Poor implementation has been implicated as a key reason for this gap.^1^

While some authors have called for more rigorous, equity focused science,^2,3^ others opine that social science theories hold the key to practical implementation.^4^ Despite this variance in opinion, there is a near consensus amongst experts that successful implementation in global health requires innovative paradigms.

Ancient Greek philosopher, Aristotle, held that knowledge exists in three levels: *Theoria*(thinking), *poiesis* (making), and *praxis* (doing).^5^ Much of the existing knowledge in implementation science has hovered around the first level (theory)^6^ with some advancement into the second level (production of models and tools). But it is increasingly recognized that there is much to be done to advance the field into ‘praxis’ (informed, committed action), which some implementation scientists have recently dubbed “Implementation Science 3.0.”^6,7^

 Our article advances the current debate by drawing attention to how the ‘art and craft’ of the industrial sector will contribute practicable lessons to building the discipline of implementation in global health beyond current ‘scientific’ methods.

## The Unique Challenge With Implementation Science in Global Health

The definitions and the scope of global health are very much under debate.^8-12^ Many scholars have viewed global health as high-income countries’ (HIC) interventions in LMICs. But some others argue that the field is one that addresses inequalities between and within countries across the world.^8,10^ A recent article prefers an alternative definition of global health as “public health everywhere.” The authors argue that global health should emphasize the applicability of a health intervention in the global context in contrast to the specific settings where it has been piloted.^10^ There is no contention that implementation gaps are significant in the field, irrespective of the definition. This is particularly true for LMICs, where contextual uncertainties abound, compounding the existing resource constraints. Our perspective focuses on addressing challenges with implementing health interventions across all jurisdictions, but with emphasis on the complexities and challenges in LMICs.

Given the complexity of global health, approaches to improve implementation must be sensitive to such complex adaptive systems.^13^ Most of the existing implementation science approaches originate from HICs, and often do not reflect the contexts, paradigms, priorities and systems operational in many LMICs.^2^ Reflecting on the implications of political dynamics on implementation, one author posits that “all the most authoritative conceptualizations mentioned here were modelled on Western-style democratic governance systems.”^14^ Thus, there is the tendency for “philosophical bias” with many frameworks, as these contributions to the science may reflect the proposer’s own perspective and contextual experiences.^15^ Yet there is a need for researchers, policy-makers, health practitioners and other actors in other contexts to be able to optimize implementation in their settings, despite how complex the setting may be. These issues around balancing contextual diversity are part of ongoing discussions on ethical considerations on global health.^16^

Paradigms, values and systems differ across nations, often shaped by their unique cultures and histories. These have implications on how health systems, policies and organizations are governed and operated. In many LMICs, the universal health coverage principles, such as equity, drive how policies and programs are designed and implemented.^3^ In contrast, it has been argued that profit making supersedes equity considerations in capitalist systems operated in countries like the United States.^17^ Contextual variances, though well recognized in the implementation science literature, are frequently not addressed by the existing tools designed for the real world. Take for example, a recent effort to optimize the consolidated framework for implementation research for use in LMICs.^18^ The authors, recognizing the limitations of the existing framework, prescribe a new domain and eleven novel constructs for inclusion to the consolidated framework for implementation research to increase its compatibility for use in LMICs. But while frameworks developed from scientific approaches such as systematic reviews may broadly indicate areas or domains to be considered by researchers, policy-makers or implementers, they are insufficient to address real world challenges in complex adaptive systems.^19^ More pragmatic approaches are needed to complement these frameworks.

LMICs continue to grapple with the problem of how to effectively design and implement policies, programs and other interventions. This challenge is compounded by the urgent need bridge the gaps in health outcomes amidst the limited resources in these countries. Most LMICs failed to meet the millennium development goals, and there are concerns that many are lagging behind in meeting targets of the sustainable development goals and universal health coverage.^20^ More practical implementation science has been advocated to accelerate the effective execution of planned strategies in this direction. But success will require LMIC actors to have contextualized tools and resources to complement the theories and frameworks contained in the current implementation science literature. As with the extant tools mostly drawn from knowledge gained from HICs, practical experiences from LMICs are invaluable in the development and application of in-grown strategies to solve local problems. A recent article demonstrates the efforts that have been made thus far in developing a framework of core competencies for implementation research in resource constrained settings.^21^ In the article, the authors underscored the need to establish proficiency levels for implementation research based on generally applicable theories for competency-based training. They further identified the lack of involvement of practitioners from LMICs in the development of the framework as a significant gap.^21^ Practical lessons from industry can bridge this gap and provide the needed competency-based training to maximize outputs from investments in health interventions.

## Practical Learnings From Industry

Global health exhibits key traits of business. There is the demand side and there is the supply side. Optimum results are achieved at equilibrium where supply meets demand such that health commodities and human resources with adequate capacity are available to provide equitable services that meet the needs of the population.^22^ Effective implementation in global health aims at attaining this equilibrium point. In addition, there are the investors (governments, donors, etc), who are concerned about value for money invested to achieve health improvements.^23^ There are also the clients (patients, communities, etc), who expect to receive quality services. Then there are the people and the organizations engaged as agents of service or product delivery. All these actors desire specific results which often differ and may conflict. For example, governments and donors often use high level results-based management indicators to measure the performance of funded initiatives. Thus, they are often concerned with feedback related to, cost-effectiveness, allocative efficiency and distribution (who gets how much of what types of services and products).^23,24^ Frontline health workers, on the other hand, are mostly concerned with clinical improvement of individual patients, who in turn are interested in having physical and financial access to quality care.^22^

Underscoring the business nature of global health now are the investments, worth billions of dollars, being made in the sector by business tycoons such as Bill Gates and Africa’s wealthiest entrepreneur, Aliko Dangote, which have only further blurred the lines between industry and the health sector.^25,26^ This convergence is further exemplified by the conscription of business management firms to support the design and implementation of several global health projects.^27^ While the effectiveness of this strategy may not yet be “scientifically” tested, and the results may vary with context, it may be worthwhile to incorporate commonsense industry principles and practices into global health learning curricula. Thus, the academic implementation science community may benefit from “art and craft” used in building successful businesses and transferring knowledge and skills across generations since ancient times.

## Getting Things Done in the Real World

Everyone desires results but not everyone is getting the kind of results they desire. The problem of implementation is not unique to the global health academic community, which has more recently been engaged in evolving ‘scientific’ strategies to address this challenge.^28^ Actors in industry have long engaged in debates around improving the art and craft of execution, that is “getting things done.”^29^ Both the academia and the business community are in agreement that implementation is a discipline, a branch of knowledge.^29,30^ But both communities seem to differ on how to approach this discipline.

On one hand, implementation scientists have evolved several strategies and frameworks, derived from empirical and theoretical evidence, in an attempt to propound theories, models and frameworks on how to get the ‘right things done’ across various contexts.^31^ On the other hand, industry leaders have often emphasized the importance of complementing scientific projections with artistic skills born out of imagination and intuition, often specific to circumstance.^32^ For example, the academic literature recommends diverse “evidence-based” strategies and frameworks, hinged on behavioral science theories, for engaging stakeholders to improve implementation.^33^ But industry actors like Jack Welch, the former chief executive officer of General Electric, believe that people management is a soft skill acquired from empirical knowledge, and “trusting your guts.”^32^ Thus, to the entrepreneur, effective implementation strategies result from skills in observation, experimentation, reflection and intuition.

Whereas science generates knowledge largely from observation and experimentation, the arts are hinged on reflection and intuition which cannot be measured scientifically, nor are do they always produce generalizable knowledge. The skillful amalgamation of these approaches yields a craft learned only through series of trial and error.^34^ Thus, like other practical skills from sports to business management, the craft of implementation is a real-world expertise developed over time through experience and learning. But this process is more effectively facilitated by mentorship and apprenticeship – a classical approach to “knowledge transfer” in industry from time immemorial.^35^ Mentorship involves guidance provided by skilled experts to support their mentees to attain their dreams and maximize their potentials. In an apprenticeship, the skilled mentors invest in replicating themselves (skills and knowledge) in their apprentices.^36^

In terms of training practitioners, critical implementation science knowledge and skills relevant to global health have been highlighted in the literature.^3^ These knowledge and skills include how to design and optimally apply strategies to improve access to quality care, how to adapt interventions and strategies to local contexts, how to scale up interventions across governance tiers, and how to plan and design interventions and strategies for sustainable implementation and impact. Whereas “applied” implementation science focused practicums, papers, and special studies have been incorporated into many academic programs,^6,37-39^ lack of sustained interaction has been identified as a key gap in many of these training programs.^39^ It is increasingly recognized that “one-off” training models are not very effective measures to improve global health practice in the long-term, thus we propose more sustainable approaches that will bridge the persisting gap between academics and real-world practitioners.

Based on the arguments highlighted above, we recommend capacity building approaches that emphasize relationship building, supportive supervision and coaching, as the fix required to move the discipline forward. One approach could be to assign global health students to mentors from the healthcare industry. Ideally, such mentors should be vastly knowledgeable and professionally versatile in the business side of health. They should also be context-literate, having a clear understanding (from long term experience) of what works, what does not work, and how things work in the specific setting of training interest. Another approach could focus on the re-design of training programs to lay more emphasis on the practical (with internships) than the theoretical components. Such models have been successfully implemented in the arts, medicine and more recent field epidemiology training programs.^35,40,41^ Two-way secondment models may also be explored in which industry actors are attached to academic institutions, while academics are seconded to industries. Researchers (or students) in industry will acquire skills in practical implementation, while quality evidence generation techniques and tools can improve industry outputs. These mentorship and apprenticeship models bring with them aspects of connoisseurship, tacit knowledge, reputation, and relationships which are important to real-world practice. This approach will prove impactful in emerging practical disciplines like implementation science, as it has in medicine,^41^ and in trans-generational business brands.^35^ In addition, a long-term effect of this relationship will be the prioritization of research and training to meet real-world needs. Thus, academia produces knowledge more relevant in real-world industry, and industry contributes more significantly to academic capacity building – blurring the age long divide between the “two communities.”^42^ This way, global health students, teachers and industry practitioners can benefit from the diffusion of knowledge and experience.

Critical participatory action research (CPAR) is another approach that can contribute to knowledge, co-develop and test practical tools in real-world context, with real world practitioners.^43^ Like traditional participatory action research, CPAR blurs the line between researchers and practitioners, (policy-makers, implementers and communities) by collective participation and action.^44^ In addition, CPAR is rooted in the critique of conventional social and action research. The approach considers action research itself a social practice — ‘a practice changing practice.’^45^ Participants in CPAR introspect individually and collectively on their own practice with the aim of changing (a) their ‘sayings,’ ‘doings’ and ‘relatings’ or interactions in their current practice with a view to understanding how these constrain or facilitate innovative ways of acting, individually and collectively.^46^

 Figure illustrates the gap between academia and industry, as well as some core competencies required for real world implementation and how CPAR, mentorship, apprenticeship can provide the needed “two-way bridge” between the two communities, with impact on the third -service users.

**Figure:**
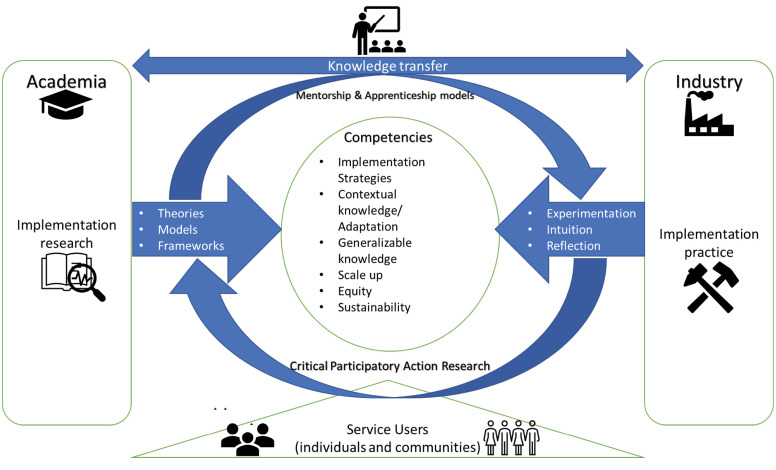
An Illustration of Proposed Capacity Building Approach.

 The academic community continues to produce knowledge on generalizable theories, frameworks and models to better implement health interventions. Industry has a long memory of what works in the real world learned through experimentation, experience, intuition and reflection. Knowledge transfer through internships and apprenticeships can bridge the gap and produce implementation leaders well equipped with the requisite competencies required in real world health systems, as well as contribute to more need-tailored knowledge generation in academia. While interactions and inputs from service users will tailor interventions to meet the quality and equity needs of individuals and communities.

## Conclusion

Incorporating approaches such as industry mentorship and apprenticeship, and CPAR into implementation science training programs can build leaders who have the capacity to produce desired results both in industry and in academia; bridging the “know-do” gap, a core pursuit of the science of implementation.^31^ There is indeed an urgency to ensure that the next-generation of implementation science experts are “oven-ready” to face the realities of practice, especially as they are coming into a real-world with less than a decade left to achieve its sustainable development goals and one fraught with uncertainties (including climate change and pandemics) for which there will be increased need for experts who can do it right. An opportunity currently exists with the recent expansion and penetration of implementation science into academic programs across the world, LMICs inclusive. As the curriculum also evolves from being largely focused on Western paradigms to being more applicable to global health contexts, incorporating apprenticeship and mentorship from the healthcare industry may just be the key to getting the right things done right in real-world health systems. Thus, advancing the field beyond *theoria* to more of *poiesis* and *praxis.*

## Ethical issues

 Not applicable.

## Competing interests

 Authors declare that they have no competing interests.

## Authors’ contributions

 EAE conceptualized the article, EAE and ABT wrote the article.

## Authors’ affiliations

^1^Department of Medicine, Faculty of Medicine and Dentistry, University of Alberta, Edmonton, AB, Canada. ^2^Department of Planning, Research and Statistics, National Primary Health Care Development Agency, Abuja, Nigeria. ^3^Department of Health Policy, London School of Economics and Political Science, London, UK.
